# Do More Screws Mean Better Stability? Four (4S) vs. Six (6S) Screws for Short-Segment Fixation in Thoracolumbar Fractures—A Systematic Review and Meta-Analysis

**DOI:** 10.3390/jcm14165672

**Published:** 2025-08-11

**Authors:** Andrea Perna, Andrea Franchini, Giuseppe Rovere, Calogero Velluto, Maria Ilaria Borruto, Laura Scaramuzzo, Felice Barletta, Luca Proietti, Franco Gorgoglione

**Affiliations:** 1 Department of Orthopaedics and Traumatology, Fondazione Casa Sollievo della Sofferenza IRCCS, 71013 San Giovanni Rotondo, Italy; a.franchini@operapdrepio.it (A.F.); f.barletta@operapadrepio.it (F.B.); f.gorgoglione@operapadrepio.it (F.G.); 2 Orthopedics and Traumatology Unit, Department of Clinical Sciences and Translational Medicine, Tor Vergata University, 00133 Rome, Italy; 3 Department of Orthopaedics and Traumatology, Fondazione Policlinico Universitario A. Gemelli IRCCS-Università Cattolica del Sacro Cuore, 00168 Rome, Italy; calogerovelluto@gmail.com (C.V.); maria.ilaria.borruto@gmail.com (M.I.B.); laura.scaramuzzo1@guest.policlinicogemelli.it (L.S.); luca.proietti@policlinicogemelli.it (L.P.)

**Keywords:** thoracolumbar fractures, short-segment fixation, pedicle screw instrumentation, intermediate screw

## Abstract

**Purpose:** Thoracolumbar burst fractures represent a significant proportion of spinal injuries, with management strategies remaining a subject of debate. While four-screw (4S) short-segment posterior fixation is commonly used, recent biomechanical studies suggest that adding pedicle screws at the fractured level (six-screw, 6S, construct) may improve stability and clinical outcomes. However, the clinical relevance of these findings remains uncertain. **Methods:** A systematic review and meta-analysis were conducted in accordance with PRISMA guidelines. Three databases (Scopus, PubMed/MEDLINE, Cochrane Library) were searched for studies comparing 4S and 6S constructs in thoracolumbar fractures. Inclusion criteria encompassed comparative clinical studies reporting perioperative, functional, and radiological outcomes. Data were extracted and analyzed using Review Manager 5.4.1, applying fixed- or random-effects models based on heterogeneity. **Results:** Twenty-two studies involving 1595 patients were included. The 6S group showed significantly improved postoperative pain scores (VAS), better short- and long-term sagittal alignment, and a lower implant failure rate. However, this technique was associated with longer operative times, increased intraoperative blood loss, and extended hospital stays. No significant differences in long-term functional disability (ODI) or infection rates were found. **Conclusions:** The addition of intermediate screws improves radiological outcomes and reduces implant failure but increases surgical burden. Further high-quality studies focusing on patient-reported outcomes and specific fracture subtypes are needed to refine clinical indications.

## 1. Introduction

Thoracolumbar spinal fractures represent the most frequently observed spinal injuries [1]. Among these, vertebral burst fractures (VBFs) account for approximately 20% of all vertebral fractures [1]. Their incidence displays a bimodal age distribution: a first peak occurs in younger individuals, typically resulting from high-energy trauma such as motor vehicle accidents or falls from height; a second peak is seen in the elderly population, usually following low-energy trauma due to underlying osteoporosis [2]. Despite the wide range of treatment options proposed over the years, the optimal management of thoracolumbar fractures remains a matter of ongoing debate. In the absence of neurological impairment or spinal instability, conservative treatment is often considered appropriate [3]. However, surgical intervention is increasingly favored. Among surgical options, posterior pedicle screw fixation is the most widely adopted technique. It serves to restore vertebral body height, correct post-traumatic kyphotic deformity, reestablish spinal stability, and indirectly decompress the spinal canal [4]. For many years, the conventional surgical approach to posterior fixation has involved a short-segment construct using four pedicle screws (4S) placed one level above and one level below the fractured vertebra. This technique has been widely adopted by spine surgeons worldwide and has generally produced acceptable outcomes. However, an increasing number of studies in the literature have reported significant limitations associated with this method, including the progressive loss of kyphotic correction, screw loosening, and a higher incidence of implant failure following short-segment fixation [1,5].

Biomechanical studies demonstrated that the addition of pedicle screws at the level of the fractured vertebra (six-screws construct, 6S) can significantly enhance construct stability, improve load distribution, and reduce stress on the screws placed in adjacent, non-fractured vertebrae [6,7,8]. These findings suggest that the six-screw construct may offer superior mechanical support compared to the traditional four-screw technique. However, despite these promising biomechanical advantages, clinical evidence remains limited and inconclusive. Several small clinical studies yielded inconsistent results, with some reporting only marginal benefits or no significant improvements in clinical outcomes [9,10]. As a result, the widespread adoption of intermediate screw placement at the fracture level remains limited, and its role in routine surgical practice continues to be debated.

This systematic review and meta-analysis were conducted to compare the clinical, functional, and radiological outcomes of four-screw (4S) vs. six-screw (6S) short-segment posterior fixation in thoracolumbar fractures. In light of conflicting clinical evidence and limited high-quality data, the study also aimed to assess perioperative parameters such as complication rates, blood loss, operative time, and length of hospital stay, to better define the potential advantages of adding intermediate screws at the fracture level.

## 2. Material and Methods

### 2.1. Study Setting and Search Strategy

**Figure 1:**
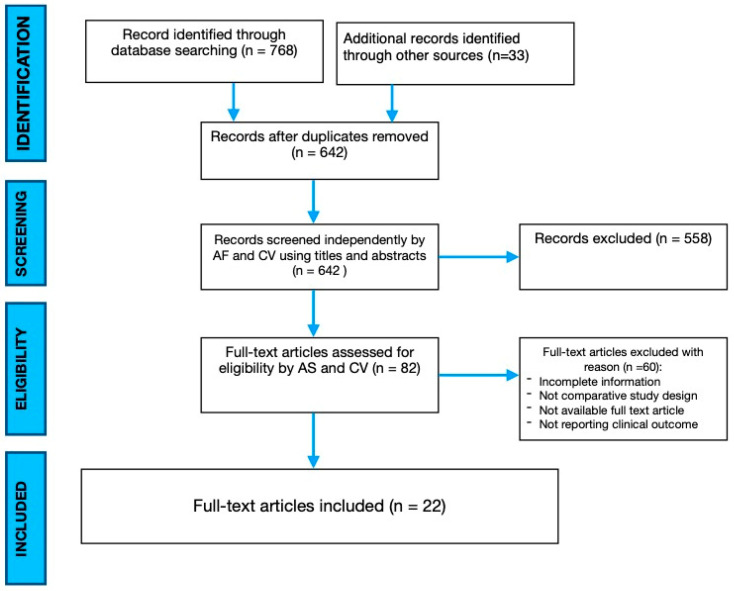
PRISMA flowchart.

This systematic review was conducted in accordance with the Preferred Reporting Items for Systematic Reviews and Meta-Analyses (PRISMA) guidelines [11]. A comprehensive literature search was carried out across three electronic databases: Scopus, the Cochrane Library, and MEDLINE via PubMed. The search strategy incorporated a combination of the following terms and their respective MeSH entries: “thoracolumbar fracture,” “intermediate screw,” “index screw,” “additional screw,” “fracture level,” and “fractured vertebra,” using logical operators “AND” and “OR” to ensure broad coverage. Reference lists of included articles were also manually screened to identify additional relevant studies. The search was updated through 1 March 2025. This review protocol, initiated on 7 February 2025, was registered with the International Prospective Register of Systematic Reviews (PROSPERO), under a temporary ID: 1034474. The PRISMA flow-chart is reported in Figure 1. 

### 2.2. Inclusion and Exclusion Criteria

This review included published full-text articles that directly compared four-screw and six-screw short-segment fixation techniques for thoracolumbar spine fractures. No restrictions were applied regarding the publication date. Articles not originally published in English were translated using artificial intelligence tools (ChatGPT, GPT-4.0, OpenAI). The accuracy of the extracted data was cross-verified multiple times by comparing translated content with corresponding information from other bibliographic sources to ensure consistency and reliability. Studies were excluded if they consisted of expert opinions, animal research, unpublished data, in vitro or cadaveric experiments, case reports, case series, letters to the editor, conference abstracts, or book chapters. Studies were eligible for inclusion only if they reported on patients with non-osteoporotic thoracolumbar burst fractures treated using short-segment constructs (four- or six-screw fixation). Studies describing osteoporotic fractures, long-segment fixation, or constructs with more than six screws were systematically excluded.

### 2.3. Review Question

The research question guiding this review was developed using the PICO framework [12], which outlines four key elements: Population (P), Intervention (I), Comparison (C), and Outcome (O). Specifically, the questions addressed were

(I) In patients with thoracolumbar vertebral fractures (P), does short-segment fixation using a six-screw construct (I) lead to superior clinical and radiological outcomes (O) compared to fixation with a four-screw construct (C)?

(II) In patients with thoracolumbar vertebral fractures (P), does short-segment fixation using a six-screw construct (I) lead to a minor complication rate (O) compared to fixation with a four-screw construct (C)?

### 2.4. Data Extraction

Title and abstract screening were conducted by two independent authors (A.F. and C.V.). Any disagreements were resolved through consensus with a third author (A.P.). Data from the selected articles were then extracted and organized into tables by the same authors. The following information was collected: demographic details, operative time, intraoperative blood loss, length of hospital stay, clinical and functional outcomes, complications, and follow-up duration.

### 2.5. Statistical Analysis

**Figure 2:**
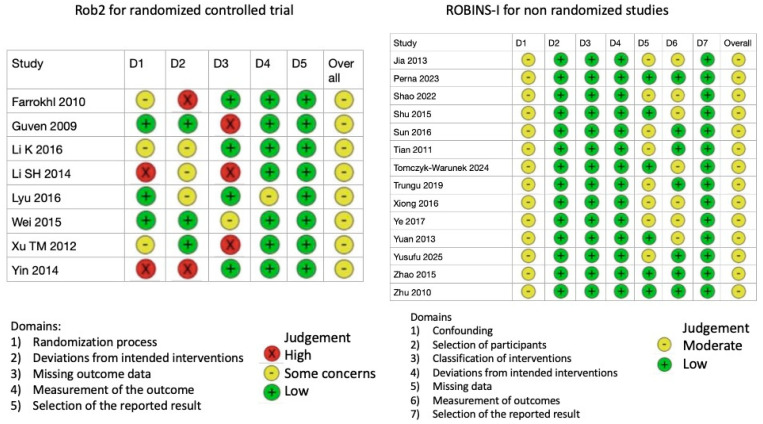
RoB2 and ROBINS-I results for all eligible, included studies. RoB2, Cochrane Risk of Bias Tool for Randomized Trials; ROBINS-I, Cochrane Risk of Bias in Non-randomized Studies of Interventions [1,10,13,14,15,16,17,18,19,20,21,22,23,24,25,26,27,28,29,30,31].

Collected data were organized using Numbers Version 14.4 software (Apple Inc., Cupertino, CA, USA). Categorical variables are presented as frequencies and percentages, while continuous variables are reported as means with standard deviations, rounded to one decimal place. Relevant measures are expressed as mean differences (MD) and odds ratios (OR) with 95% confidence intervals (CIs). A Forest plot was used to visually present the outcomes. Heterogeneity across studies was assessed using the χ^2^ test, and the I^2^ statistic was calculated to estimate the proportion of variation between studies. An I^2^ value greater than 50% was considered indicative of substantial heterogeneity. If substantial heterogeneity was detected, a random-effects model was applied; otherwise, a fixed-effects model was used. The quality of the included studies was assessed using the Cochrane Collaboration’s Tool. A summary table of risk-of-bias assessments using the Cochrane ROBINS-I tool for non-randomized studies and the RoB 2 tool for randomized trials was reported in Figure 2. Statistical analysis and Forest plot generation were performed using Review Manager Version 5.4.1 (Cochrane Collaboration, Software Update, Oxford, UK).

## 3. Results

### 3.1. Study Selection and Characteristic

The initial electronic literature search identified a total of 791 records. After removing duplicates, 642 studies remained for title and abstract screening. Of these, 82 articles were deemed potentially eligible and assessed in full text, while the rest were excluded as unrelated to the topic. Following full-text evaluation, 60 studies were excluded due to the lack of full-text availability, incomplete datasets, absence of clinical outcomes, or non-comparative study designs. As a result, 22 studies met the inclusion criteria and were incorporated into the final analysis [1,9,10,13,14,15,16,17,18,19,20,21,22,23,24,25,26,27,28,29,30,31]. Among these, 12 were prospective clinical trials and 10 were retrospective comparative epidemiological studies. A total of 1595 patients were included in the meta-analysis, comprising 767 individuals treated with a six-screw (6S) construct and 828 treated with a four-screw (4S) construct. The publication bias was analyzed, according to the meta-analysis of observational studies in epidemiology (MOOSE) criteria, by creating a funnel plot for each outcome analyzed, analyzing its asymmetry [32]. The PRISMA flow chart is reported in Figure 1. A summary of findings is reported in Table 1. A comprehensive summary of all included studies, detailing study design, sample size, and the patients included in each group, is provided in Table 2.

### 3.2. General Data

**Figure 3:**
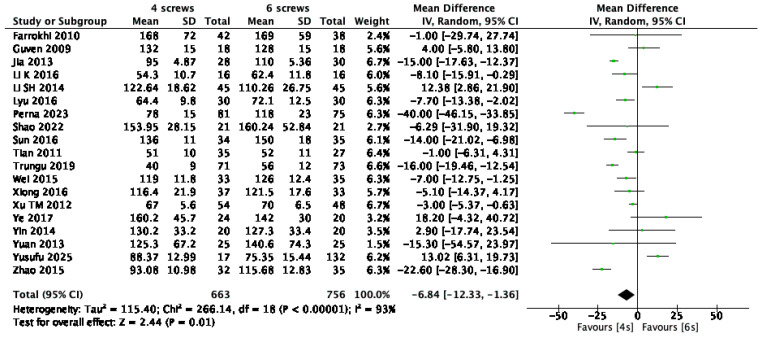
Forest plot comparing operation time for 6S vs. 4S. CI confidence interval, IV inverse variance, SD standard deviation [1,9,10,13,14,15,16,17,18,20,21,22,23,24,25,26,27,28,29].

Nineteen studies [1,9,10,13,14,15,16,17,18,20,21,22,23,24,25,26,27,28,29], involving a total of 1419 patients, reported data on operative time. The pooled analysis demonstrated that the addition of intermediate screws significantly increased the duration of surgery, with a mean difference of 6.84 min (95% CI: –12.22 to –1.26; *p* = 0.01; I^2^ = 93%) (Figure 3).

**Figure 4:**
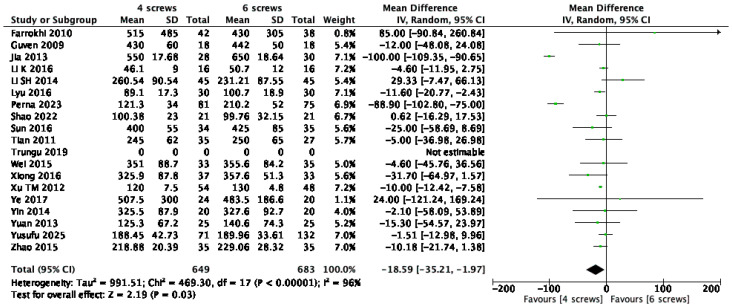
Forest plot comparing intraoperative blood loss for 6S vs. 4S. CI confidence interval, IV inverse variance, SD standard deviation [1,9,10,13,14,15,16,17,18,20,22,23,24,25,26,27,28,29].

Eighteen studies [1,9,10,13,14,15,16,17,18,20,22,23,24,25,26,27,28,29], including 1332 patients, provided data on intraoperative blood loss. The meta-analysis revealed a statistically significant mean difference of 18.59 mL in favor of the four-screw construct (95% CI: –35.2 to –1.97; *p* < 0.03; I^2^ = 96%) (Figure 4).

**Figure 5:**
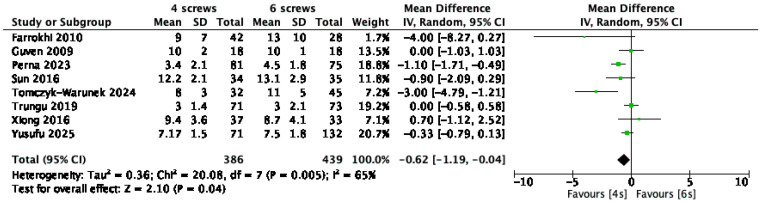
Forest plot comparing hospital length of stay for 6S vs. 4S. CI confidence interval, IV inverse variance, SD standard deviation [1,9,10,13,21,23,28,31].

Eight studies [1,9,10,13,21,23,28,31], comprising a total of 825 patients, reported hospital length of stay (LOS). The analysis indicated a significantly shorter hospital stay in the four-screw group, with a mean difference of 0.62 days (95% CI: –1.19 to –0.04; *p* < 0.04; I^2^ = 65%) (Figure 5).

### 3.3. Functional Outcomes

**Figure 6:**
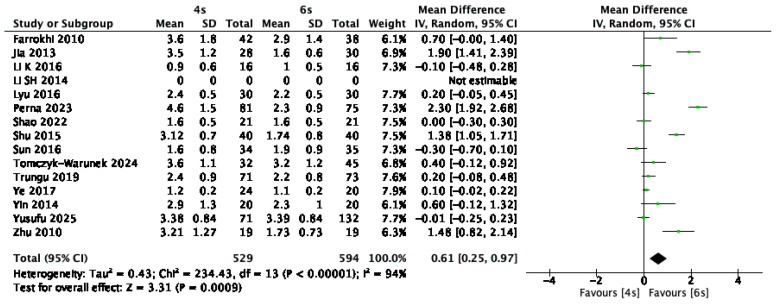
Forest plot comparing visual analog scale for 6S vs. 4S. CI confidence interval, IV inverse variance, SD standard deviation [1,9,13,14,15,16,17,18,19,21,25,26,28,30,31].

Visual Analogue Scale (VAS) scores were reported in 14 of the included studies [1,9,13,14,15,16,17,18,19,21,25,26,28,30,31], encompassing a total of 1123 patients. VAS assessments were performed at follow-up intervals of at least three months postoperatively. The pooled analysis revealed a statistically significant reduction in pain levels favoring the six-screw construct, with a mean difference of 0.61 points (95% CI: 0.25 to 0.97; *p* < 0.03; I^2^ = 94%) (Figure 6).

**Figure 7:**
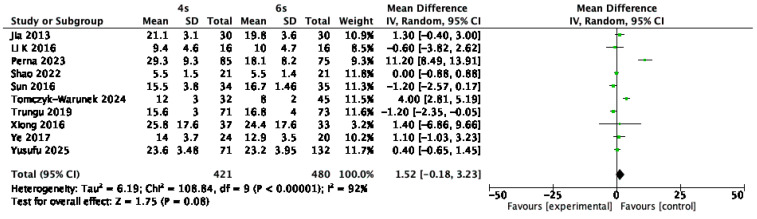
Forest plot comparing Oswestry Disability Index for 6S vs. 4S. CI confidence interval, IV inverse variance, SD standard deviation [1,9,14,15,18,21,23,25,28,31].

Data on the Oswestry Disability Index (ODI) were extracted from 10 studies, involving 901 patients [1,9,14,15,18,21,23,25,28,31]. ODI scores were collected at a minimum follow-up of one year after surgery. The meta-analysis demonstrated no statistically significant difference between the six-screw and four-screw constructs, with a mean difference of 1.52 points (95% CI: –0.18 to 3.23; *p* = 0.08; I^2^ = 92%) (Figure 7).

### 3.4. Radiological Data

**Figure 8:**
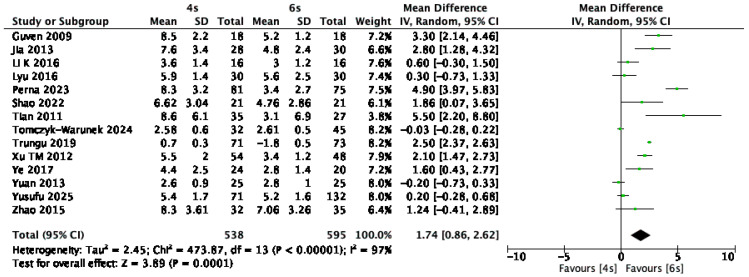
Forest plot comparing short-term postoperative Cobb angle for 6S vs. 4S. CI confidence interval, IV inverse variance, SD standard deviation [1,10,14,15,17,18,20,21,24,25,27,28,29,31].

Fourteen studies [1,10,14,15,17,18,20,21,24,25,27,28,29,31], including a total of 1133 patients, reported short-term postoperative Cobb angles, assessed between one week and one month after surgery. The pooled data demonstrated that patients treated with the six-screw construct achieved significantly improved short-term sagittal alignment, with a mean difference of 1.74° in Cobb angle favoring the 6S group (95% CI: 0.86 to 2.62; *p* < 0.01; I^2^ = 87%; *p* for heterogeneity = 0.0001) (Figure 8).

**Figure 9:**
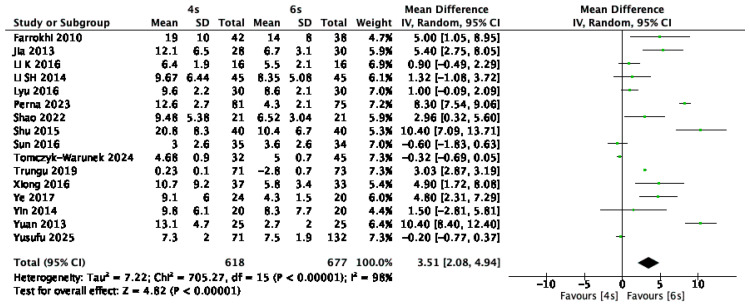
Forest plot comparing long-term postoperative Cobb angle for 6S vs. 4S. CI confidence interval, IV inverse variance, SD standard deviation [1,9,13,14,15,16,17,18,20,21,24,25,27,28,29,31].

Long-term postoperative Cobb angles—measured at least one year following surgery—were reported in 16 studies [1,9,13,14,15,16,17,18,20,21,24,25,27,28,29,31], involving a total of 1295 patients. The six-screw construct was associated with significantly better maintenance of sagittal alignment over time, with a pooled mean difference of 3.51° compared to the four-screw group (95% CI: 2.08 to 4.94; *p* < 0.00001; I^2^ = 98%) (Figure 9).

### 3.5. Complications

**Figure 10:**
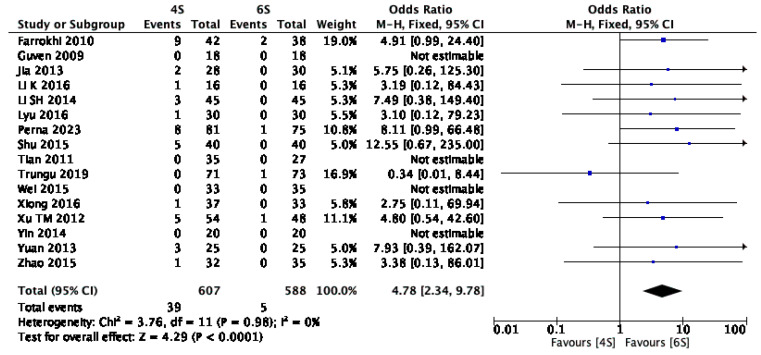
Forest plot comparing implant failure for 6S vs. 4S. CI confidence interval, M-H Mantel–Haenszel, SD standard deviation [1,10,13,14,15,16,17,19,20,21,22,23,24,26,27,29].

Sixteen studies [1,10,13,14,15,16,17,19,20,21,22,23,24,26,27,29], encompassing a total of 1195 patients and an average follow-up duration of approximately two years, reported data on postoperative implant failure. The pooled analysis demonstrated a significantly lower incidence of implant failure in the six-screw construct group, with an odds ratio (OR) of 4.78 (95% CI: 2.34–9.78; *p* < 0.0001; I^2^ = 0%) (Figure 10).

**Figure 11:**
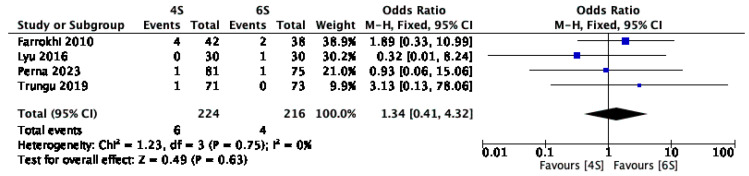
Forest plot comparing postoperative infection for 6S vs. 4S. CI confidence interval, M-H Mantel-Haenszel, SD standard deviation [1,13,17,21].

Only four studies [1,13,17,21], involving 440 patients, provided information on postoperative infection rates. The fixed-effect model revealed no statistically significant difference between the two groups regarding infection (OR: 1.34; 95% CI: 0.41–4.32; *p* = 0.63; I^2^ = 0%) (Figure 11).

Additional postoperative complications were sporadically reported across various studies but were too infrequent to support a pooled analysis. These included four cases of deep vein thrombosis (one in the 4S group and three in the 6S group), and fifteen cases of chronic lower-back pain (eleven in the 4S group and four in the 6S group).

### 3.6. Subgroup Analysis

Among the studies included in our analysis, five [15,17,18,19,21] utilized a percutaneous approach for short-segment fixation. When compared to studies employing open surgical techniques, the results were largely consistent. These percutaneous series demonstrated that six-screw constructs (6S) were associated with superior correction of post-traumatic kyphosis, improved radiographic alignment parameters, and a reduced incidence of hardware failure, mirroring the overall trends observed in the pooled data. This consistency across surgical approaches reinforces the mechanical advantage of including pedicle screws at the fracture level, regardless of whether fixation is performed percutaneously or via an open approach.

Additionally, one study [1] provided a direct comparison between open six-screw constructs and percutaneous four-screw constructs. Despite the difference in surgical technique, the outcomes reported were in line with the broader findings of our meta-analysis—favoring the six-screw configuration in terms of sagittal correction and construct durability. These findings suggest that the biomechanical benefits of fracture-level instrumentation may outweigh the influence of the surgical approach itself, further validating the robustness and clinical applicability of our results.

### 3.7. Review Questions

Response to question I: In patients with thoracolumbar vertebral fractures, the six-screw construct is associated with superior radiological outcomes and better postoperative pain control compared to the four-screw technique. Although no significant differences were found in long-term functional recovery, the improvements in sagittal alignment and pain reduction suggest a higher overall clinical efficacy of the six-screw configuration. However, these advantages must be weighed against the increased operative time, greater intraoperative blood loss, and longer hospital stay. Therefore, the choice of construct should be tailored to the patient’s clinical profile and therapeutic goals, with the six-screw technique being particularly suitable when enhanced stability and the better preservation of spinal alignment are priorities.

Response to question II: The six-screw construct also demonstrates superiority in terms of mechanical safety, showing a significantly lower risk of implant failure over mid-term follow-up. This structural benefit is achieved without an increase in infection rates or systemic complications, indicating that the addition of intermediate screws is an effective strategy to enhance construct stability without compromising tolerability. In summary, the six-screw configuration appears to be a safer and more reliable surgical option for managing thoracolumbar fractures, particularly in cases where long-term implant integrity is a critical concern.

## 4. Discussion

The surgical treatment of thoracolumbar fractures aims primarily to decompress neural structures when necessary, restore physiological sagittal alignment, and facilitate early mobilization. Despite advances in spinal instrumentation, the optimal surgical approach for unstable compression and burst fractures remains a subject of continued debate.

Traditionally, long-segment posterior fixation (LSPF), involving pedicle screw instrumentation spanning two or more levels above and below the injury, has been employed to achieve satisfactory alignment and promote fracture healing. However, this approach is not without drawbacks. The extensive fusion required in LSPF significantly limits spinal mobility and is associated with prolonged operative times, greater intraoperative blood loss, and longer hospital stays [8,33,34].

In response to these limitations, short-segment posterior fixation (SSPF) emerged as a less invasive alternative. This technique uses a four-screw (4S) construct with pedicle screws placed one level above and below the fracture, sparing motion segments and aiming to reduce surgical morbidity [35]. However, in cases where multiple spinal columns are compromised, 4S constructs are subjected to high biomechanical stress, increasing the risk of hardware failure and loss of correction [8,33].

To overcome these limitations, a modified version of SSPF—six-screw fixation (6S)—was introduced, which includes screws inserted directly into the fractured vertebra. First proposed by Dick et al. in 1994 [4], this construct enhances stability by distributing mechanical loads across three vertebrae, thereby reinforcing the injured segment and reducing stress on adjacent levels [1,8,36].

Biomechanical studies consistently demonstrate the superiority of the 6S configuration over the 4S model in resisting axial, torsional, and shear forces. This improved stability translates into the more effective restoration of vertebral body height and sustained correction of kyphotic deformity—key goals in the management of thoracolumbar trauma. Moreover, the 6S approach has been associated with a lower incidence of hardware-related complications, such as screw loosening and implant failure, particularly in high-load fracture patterns [6,7,8,36].

Despite its mechanical advantages, clinical evidence comparing 6S and 4S constructs has yielded mixed results. In this systematic review and meta-analysis, the 6S technique was associated with significantly better radiographic outcomes, particularly in maintaining postoperative Cobb angles over time. This suggests a reduced risk of delayed kyphotic collapse, a complication known to negatively impact long-term function and quality of life [1,4].

Pain outcomes, as assessed by the Visual Analog Scale (VAS), also favored the 6S group, supporting the hypothesis that increased construct stability leads to better early symptomatic relief. However, the Oswestry Disability Index (ODI), a measure of functional recovery, did not differ significantly between the two groups. Regarding the lack of significant difference in ODI scores between the 4S and 6S groups, we acknowledge that this finding warrants further context. Several factors may contribute to this result. First, the relatively short follow-up duration in many of the included studies may limit the ability to capture long-term functional improvements, especially in a young and active patient population. Second, the ODI—while widely used—may have limited sensitivity in detecting subtle clinical differences in patients recovering from thoracolumbar trauma, particularly when radiographic and symptomatic outcomes show divergence. Lastly, a potential ceiling effect may play a role, as patients with high baseline functional capacity may achieve near-maximal scores postoperatively regardless of construct type [1,4].

Perioperative metrics such as operative time, blood loss, and hospital stay were slightly elevated in the 6S group but remained clinically insignificant. The average increase in surgical duration was less than 10 min, and additional blood loss averaged under 20 mL. Similarly, hospitalization was extended by less than one day on average. These modest increases do not offset the potential benefits of improved stabilization and reduced complication rates [13,14,15,16].

Importantly, the 6S approach was associated with a lower rate of implant failure, reinforcing its biomechanical resilience. The addition of screws at the fracture level did not result in a higher incidence of infection or other common complications. Rates of thromboembolic events and chronic back pain were low and comparable between groups, indicating that the enhanced construct rigidity does not compromise safety [1,21,31].

In summary, the 6S short-segment construct offers distinct biomechanical and radiographic advantages over the traditional 4S model, with improved pain outcomes and a lower risk of implant-related complications. Although functional differences remain equivocal, the 6S technique presents a compelling alternative in the surgical management of thoracolumbar fractures, particularly in cases with high mechanical demands and multicolumn instability.

### Limitations

This systematic review and meta-analysis present several limitations that should be acknowledged. First, no subgroup analysis was performed to differentiate outcomes between open and percutaneous short-segment fixations, which may influence both clinical and radiological results. Second, many of the included studies reported a relatively low number of patients, which limits the statistical power of individual comparisons and may introduce variability in pooled estimates. Third, although this analysis included comparative studies, a considerable proportion were non-randomized and retrospective in nature, increasing the potential risk of selection bias and reducing the overall quality of evidence. Additionally, the heterogeneity among studies was substantial for several outcomes, likely due to differences in surgical techniques, implant systems, surgeon experience, and follow-up duration. Additionally, although several outcomes showed statistically significant differences—such as operative time, intraoperative blood loss, and hospital stay—the absolute magnitudes of these differences were relatively small. For instance, the mean differences of 6.84 min in operative time, 18.59 mL in blood loss, and 0.62 days in hospitalization may not reflect clinically meaningful advantages in routine practice. These findings, while statistically robust, should be interpreted with caution and within the broader context of patient-centered outcomes and surgical decision-making.

Language restrictions may have also led to selection bias, although efforts were made to include and translate non-English papers using artificial intelligence tools validated against reference sources. The inclusion of non-English articles may have introduced selection bias; non-English studies were included and translated using AI-based tools, with all translations verified by bilingual reviewers. However, the possibility of semantic inaccuracies remains.

## 5. Conclusions

Our review and meta-analysis suggest that adding intermediate screws at the fracture level during the short-segment posterior fixation for surgical treatment of thoracolumbar fixation may enhance surgical outcomes leading to less postoperative pain, improved spinal alignment, and a reduced risk of implant failure. However, these benefits come at the expense of longer procedures and greater blood loss. While the findings point toward a potential clinical advantage, the actual impact appears modest, and the quality of evidence remains limited. To truly define the role of intermediate screws, future studies should shift focus toward patient-centered outcomes and explore which fracture subtypes stand to benefit most from this technique.

## Figures and Tables

**Table 1:** Summary of main findings comparing 4S and 6S constructs.

Outcome	4-Screw (4S)	6-Screw (6S)	Difference/Observation
**Radiological Outcome (Cobb angle)**	Less effective sagittal alignment correction	Superior correction and maintenance over time	6S significantly better both short- and long-term
**Vertebral Height Restoration**	Moderate	More effective	6S construct better preserves vertebral height
**Pain (VAS Score)**	Higher VAS scores at follow-up	Lower VAS scores at follow-up	6S associated with better pain control
**Functional Outcome (ODI)**	Comparable improvement	Comparable improvement	No statistically significant difference
**Implant Failure Rate**	Higher risk	Lower risk	6S more stable, fewer mechanical failures
**Infection Rate**	Similar incidence	Similar incidence	No significant difference
**Operative Time**	Shorter	Slightly longer (+6.84 min)	Statistically significant but clinically negligible
**Blood Loss**	Lower	Slightly higher (+18.59 mL)	Minimal, not clinically relevant
**Hospital Stay**	Slightly shorter	Slightly longer (+0.62 days)	Difference small, unlikely to affect costs
**Complications (e.g., DVT, chronic pain)**	Rare, no clear trend	Rare, no clear trend	No significant difference

**Table 2:** Number of patients and study design.

Study	N of Patients	6S	4S	Study Design	Note
Farrokhi 2010 [13]	80	42	38	RCT	Open
Guven 2009 [10]	36	18	18	RCT	Open, monoaxial screws
Jia 2013 [14]	58	30	28	Prospective study	Open
Li K 2016 [15]	32	16	16	Prospective randomized study	Percutaneous
Li SH 2014 [16]	90	45	45	Prospective randomized study	Open
Lyu 2016 [17]	60	30	30	RCT	Percutaneous
Perna 2023 [1]	156	75	81	Retrospective cohort study	4S Percutaneous, 6S Open
Shao 2022 [18]	42	21	21	Retrospective cohort study	Percutaneous
Shu 2015 [19]	80	40	40	Prospective study	Percutaneous
Sun 2016 [9]	69	35	34	Retrospective cohort study	Open
Tian 2011 [20]	62	27	35	Retrospective cohort study	Open
Tomczyk-Warunek 2024 [31]	77	45	32	Prospective study	Open
Trungu 2019 [21]	144	73	71	Retrospective cohort study	Percutaneous
Wei 2015 [22]	68	35	33	RCT	Open
Xiong 2016 [23]	70	33	37	Prospective study	Open
Xu TM 2012 [24]	102	48	54	RCT	Open
Ye 2017 [25]	44	20	24	Retrospective cohort study	Open
Yin 2014 [26]	40	20	20	RCT	Open
Yuan 2013 [27]	50	25	25	Prospective study	Open
Yusufu 2025 [28]	203	132	71	Retrospective cohort study	Open
Zhao 2015 [29]	67	35	32	Retrospective cohort study	Open
Zhu 2010 [30]	38	19	19	Prospective study	Open
Total	1668	864	804		

## References

[1] Perna A., Franchini A., Gorgoglione F.L., Barletta F., Moretti B., Piazzolla A., Bocchi M.B., Velluto C., Tamburrelli F., Proietti L. (2024). Short-segment percutaneous fusion versus open posterior fusion with screw in the fractured vertebra for thoracolumbar junction burst vertebral fracture treatment. J. Neurosci. Rural. Pract..

[2] Bensch F.V., Koivikko M.P., Kiuru M.J., Koskinen S.K. (2006). The incidence and distribution of burst fractures. Emerg. Radiol..

[3] Denis F., Armstrong G.W., Searls K., Matta L. (1984). Acute thoracolumbar burst fractures the absence of neurologic deficit. A comparison between operative and nonoperative treatment. Clin. Orthop. Relat. Res..

[4] Kapoen C., Liu Y., Bloemers F.W., Deunk J. (2020). Pedicle screw fixation of thoracolumbar fractures: Conventional short segment versus short segment with intermediate screws at the fracture level—A systematic review and meta-analysis. Eur. Spine J..

[5] Perna A., Santagada D.A., Bocchi M.B., Zirio G., Proietti L., Tamburrelli F.C., Genitiempo M. (2021). Early loss of angular kyphosis correction in patients with thoracolumbar vertebral burst (A3–A4) fractures who underwent percutaneous pedicle screws fixation. J. Orthop..

[6] Bolesta M.J., Caron T., Chinthakunta S.R., Vazifeh P.N., Khalil S. (2021). Pedicle screw instrumentation of thoracolumbar burst fractures: Biomechanical evaluation of screw configuration with pedicle screws at the level of the fracture. Int. J. Spine Surg..

[7] Baaj A.A., Reyes P.M., Yaqoobi A.S., Uribe J.S., Vale F.L., Theodore N., Sonntag V.K.H., Crawford N.R. (2011). Biomechanical advantage of the index-level pedicle screw in unstable thoracolumbar junction fractures. J. Neurosurg. Spine.

[8] Norton R.P., Milne E.L., Kaimrajh D.N., Eismont F.J., Latta L.L., Williams S.K. (2014). Biomechanical analysis of four- versus six-screw constructs for short-segment pedicle screw and rod instrumentation of unstable thoracolumbar fractures. Spine J..

[9] Sun C., Guan G., Liu X., Zhang H., Wang B. (2016). Comparison of short-segment pedicle fixation with versus without inclusion of the fracture level in the treatment of mild thoracolumbar burst fractures. Int. J. Surg..

[10] Guven O., Kocaoglu B., Bezer M., Aydin N., Nalbantoglu U. (2009). The use of screw at the fracture level in the treatment of thoracolumbar burst fractures. J. Spinal Disord. Tech..

[11] Moher D., Liberati A., Tetzlaff J., Altman D.G., PRISMA Group (2009). Preferred reporting items for systematic reviews and meta-analyses: The PRISMA statement. PLoS Med..

[12] Schardt C., Adams M.B., Owens T., Keitz S., Fontelo P. (2007). Utilization of the PICO framework to improve searching PubMed for clinical questions. BMC Med. Inform. Decis. Mak..

[13] Farrokhi M.R., Razmkon A., Maghami Z., Nikoo Z. (2010). Inclusion of the fracture level in short segment fixation of thoracolumbar fractures. Eur. Spine J..

[14] Jia Q.Y., Wang L., Yu Y., Guo W.G., Yang N. (2014). Clinical comparative study of short-segment fixation for thoracolumbar burst fracture via or not via injured vertebra. Chin. J. Bone Jt. Inj..

[15] Li K., Li Z., Ren X., Xu H., Zhang W., Luo D., Ma J. (2016). Effect of the percutaneous pedicle screw fixation at the fractured vertebra on the treatment of thoracolumbar fractures. Int. Orthop..

[16] Li S.H., Tang Y.Z., Yang L.Q., Wang C.Q., Li H.Y., Wang X.J. (2014). A comparative study of the efficacy of through injured vertebra method and across the injured vertebra method in treatment of thoracolumbar spine fracture. J. Bethune Med. Sci..

[17] Lyu J., Chen K., Tang Z., Chen Y., Li M., Zhang Q. (2016). A comparison of three different surgical procedures in the treatment of type A thoracolumbar fractures: A randomized controlled trial. Int. Orthop..

[18] Shao X., Peng P., Yang P., Xu T., Liu Z., Hua X., Zhu X., Qian Z., Yang H., Mao H. (2022). A retrospective comparative study of clinical efficacy of percutaneous short segment pedicle screw fixation with or without screwing of the fractured vertebra with O-arm navigation. BMC Musculoskelet. Disord..

[19] Shu J.C., Qiu Z.J., Shi K.C., Wang L.E., Pei F.Q., Zhu Y. (2015). Clinical investigation of short-segment pedicle fixation at fracture level in the treatment of thoracolumbar fractures. Chin. J. Bone Jt. Inj..

[20] Tian J., Wang L., Xia T., Liu C., Zhao Q., Dong S. (2011). Posterior short-segmental fixation combined with intermediate screws vs conventional intersegmental fixation for monosegmental thoracolumbar fractures. Orthopedics.

[21] Trungu S., Forcato S., Bruzzaniti P., Fraschetti F., Miscusi M., Cimatti M., Raco A. (2019). Minimally invasive surgery for the treatment of traumatic monosegmental thoracolumbar burst fractures: Clinical and radiologic outcomes of 144 patients with a 6-year follow-up comparing two groups with or without intermediate screw. Clin. Spine Surg..

[22] Wei M., Liu Z., Liao W.B., Zeng Y.G., Wu G.H. (2015). Pedicle screw fixation through the fracture vertebra with across the fracture level for thoracolumbar fractures. Guangxi Med. J..

[23] Xiong J., Song Z.H. (2016). Compare the short segment internal fixation at fracture vertebra with not at fracture level for treatment of thoracolumbar fractures. J. Pediatr. Orthop..

[24] Xu T.M., Xu Y.Q., Chen J.M., Zhang C.C., Li Y., Li Z.Q. (2012). Short-segment pedicle screw fixation with screw placed at single injured vertebrae combined with transpedicular intracorporeal grafting for thoracolumbar fractures. Chin. J. Bone Jt. Inj..

[25] Ye C., Luo Z., Yu X., Liu H., Zhang B., Dai M. (2017). Comparing the efficacy of short-segment pedicle screw instrumentation with and without intermediate screws for treating unstable thoracolumbar fractures. Medicine.

[26] Yin F., Sun Z., Yin Q., Liu J., Gu S., Zhang S. (2014). A comparative study on treatment of thoracolumbar fracture with injured vertebra pedicle instrumentation and cross segment pedicle instrumentation. Chin. J. Reparative Reconstr. Surg..

[27] Yuan Z.F., Shao B., Zeng J.P. (2013). Pedicle screw fixation at the fracture vertebrae in the treatment of thoracolumbar fractures. J. Spine Surg..

[28] Yusufu A., Abulaiti A., Haibier A., Ma J., Ma Y. (2025). Analysis of the short-term effect of three different level pedicle screws in the treatment of thoracolumbar type A fractures. J. Orthop. Surg. Res..

[29] Zhao Q.M., Gu X.F., Yang H.L., Liu Z.T. (2015). Surgical outcome of posterior fixation, including fractured vertebra, for thoracolumbar fractures. Neurosciences.

[30] Zhu Y.R., Ye X.J., Yu J.M., Jiang Y.Q., Wang H.X., Fan C.Q., Lui H., Xu G. (2010). Posterior short-segment pedicle screw fixation at the injured level for thoracolumbar spine fractures. Chin. J. Traumatol..

[31] Tomczyk-Warunek A., Kłapeć M., Blicharski R., Dresler S., Sowa I., Gieleta A.W., Skrzypek T., Lis M., Kazimierczak W., Blicharski T. (2024). Comparison of methods for short-segment posterior stabilization of lumbar spine fractures and thoracolumbar junction. J. Clin. Med..

[32] Stroup D.F., Berlin J.A., Morton S.C., Olkin I., Williamson G.D., Rennie D., Moher D., Becker B.J., Sipe T.A., Thacker S.B. (2000). Meta-analysis of observational studies in epidemiology: A proposal for reporting. Meta-analysis Of Observational Studies in Epidemiology (MOOSE) group. JAMA.

[33] McLain R.F. (2006). The biomechanics of long versus short fixation for thoracolumbar spine fractures. Spine.

[34] An H.S., Singh K., Vaccaro A.R., Wang G., Yoshida H., Eck J., McGrady L., Lim T.H. (2004). Biomechanical evaluation of contemporary posterior spinal internal fixation configurations in an unstable burst-fracture calf spine model: Special references of hook configurations and pedicle screws. Spine.

[35] Tezeren G., Kuru I. (2005). Posterior fixation of thoracolumbar burst fracture: Short-segment pedicle fixation versus long-segment instrumentation. J. Spinal Disord. Techniques.

[36] Mahar A., Kim C., Wedemeyer M., Mitsunaga L.B., Odell T.B., Johnson B., Garfin S. (2007). Short-segment fixation of lumbar burst fractures using pedicle fixation at the level of the fracture. Spine.

